# HARMONIZATION OF LATER-LIFE COGNITIVE FUNCTION ACROSS POPULATION-BASED COHORT STUDIES: RESULTS FROM HRS AND NHATS

**DOI:** 10.1093/geroni/igae098.2654

**Published:** 2024-12-31

**Authors:** Diefei Chen, Vishaldeep Sekhon, David Roth, Christopher Kaufmann, Alden Gross

**Affiliations:** Johns Hopkins University, Hanover, Maryland, United States; Johns Hopkins University, Baltimore, Maryland, United States; Johns Hopkins University, Baltimore, Maryland, United States; University of Florida College of Medicine, Gainesville, Florida, United States; Johns Hopkins Bloomberg School of Public Health, Baltimore, Maryland, United States

## Abstract

Population-based cohort studies offer unique opportunities to study characteristics of people at greatest risk for cognitive decline. Harmonizing large, well-characterized cohorts powers investigations into subgroups or rare exposures/outcomes in their associations with cognitive decline. We derived a harmonized cognition score based on cognitive tests from the Health and Retirement Study (HRS) and the National Health and Aging Trends Study (NHATS), two of the largest nationally representative cohorts of older adults in the US. Using an item banking approach based on item response theory, we leveraged tests conducted in 2012-2018 Core interviews of two studies to leverage both common (i.e. anchors) and unique items to statistically harmonize a general cognition factor score. We evaluated concurrent and predictive criterion validity of the harmonized scores by 1) cross-sectionally comparing scores at baseline (year 2012) with algorithmically defined cognitive impairment status and demographics, and 2) longitudinally assessing associations between demographics and established risk factors for cognitive decline with cognitive trajectories using mixed effects models. Cross-sectionally, the harmonized score was lowest among dementia/probable dementia group (mean = -1.84, sd = 0.75). Longitudinally, cognitive decline was steeper for participants reporting hypertension (b = -0.04, se = 0.002), stroke (b = -0.06, se = 0.004), or diabetes (b = -0.04, se = 0.003) compared to those without. Inferences were similar in each study separately. This harmonized score using data from HRS and NHATS had strong criterion validity, and can be a resource for aging researchers to examine the rare factors associated with cognitive performance in diverse populations.

